# Blockade of tumor-derived colony-stimulating factor 1 (CSF1) promotes an immune-permissive tumor microenvironment

**DOI:** 10.1007/s00262-023-03496-2

**Published:** 2023-07-28

**Authors:** Maria del Mar Maldonado, Jeffrey Schlom, Duane H. Hamilton

**Affiliations:** Center for Immuno-Oncology, Center for Cancer Research, National Cancer Institute, National Institutes of Health, Bethesda, MD 20892 USA

**Keywords:** CSF1, CSF1R, Tumor-associated macrophages, Cancer vaccines, Epitope spreading

## Abstract

The macrophage colony-stimulating factor 1 (CSF1) is a chemokine essential for the survival, proliferation, and differentiation of mononuclear phagocytes from hemopoietic stem cells. In addition to its essential physiological role in normal tissues, the CSF1/CSF1 receptor axis is known to be overexpressed in many tumor types and associated with poor prognosis. High levels of CSF1 within the tumor microenvironment have been shown to recruit and reeducate macrophages to produce factors that promote tumor invasiveness and accelerate metastasis. In this study, we demonstrate, for the first time, that treating established syngeneic murine colon and breast carcinoma tumors with a CSF1R-blocking antibody also promotes the expansion of neoepitope-specific T cells. To assess the role of tumor-derived CSF1 in these model systems, we generated and characterized CSF1 CRISPR-Cas9 knockouts. Eliminating tumor-derived CSF1 results in decreased tumor growth and enhanced immunity against tumor-associated neoepitopes, potentially promoting an immune permissive tumor microenvironment in tumor-bearing mice. The combination of neoepitope vaccine with anti-PDL1 in the MC38 CSF1−/− tumor model significantly decreased tumor growth in vivo. Moreover, anti-CSF1R therapy combined with the adeno-TWIST1 vaccine resulted in tumor control, decreased metastasis, and a synergistic increase in CD8 T cell infiltration in 4T1 mammary tumors. Analysis of the tumor microenvironment demonstrated greater CD8 T cell infiltration and a reduction in tumor-associated macrophages following CSF1R inhibition in both tumor models. Our findings thus add to the therapeutic potential of CSF1 targeting agents by employing combinations with vaccines to modulate anti-neoepitope responses in the tumor microenvironment.

**Supplementary Information:**

The online version contains supplementary material available at 10.1007/s00262-023-03496-2.

## Introduction

Tumor-associated macrophages (TAMs) are crucial components of the tumor microenvironment and often comprise nearly half of the tumor mass. Previous studies have associated the presence of TAMs with poor prognosis and tumor progression [1–7]. TAMs are thought to enhance tumor cell proliferation, promote angiogenesis, and contribute to tumor cell invasion by mediating immunosuppression in the tumor microenvironment through the recruitment of T regulatory cells (Tregs), which results in T cell inhibition and immune escape [8]. Moreover, TAMs also promote an immunosuppressive microenvironment through the secretion of cytokines such as the colony-stimulating factor-1 (CSF1).

CSF1, also known as macrophage colony-stimulating factor (M-CSF), is essential in the survival and differentiation of myeloid precursors into macrophages. It is systemically expressed and a ligand of the CSF1 receptor (CSF1R) along with interleukin-34 (IL-34). CSF1R signaling drives the production and differentiation of monocytes, circulating and tissue-resident macrophages and, as a result, it is involved in processes such as tissue inflammation, repair, and homeostasis [9]. Nonetheless, CSF1 is also a major chemoattractant that regulates the production, survival, and recruitment of TAMs to the tumor microenvironment [10].

Overexpression of CSF1 has been correlated with poor prognosis in breast, prostate, and ovarian cancer patients and has been shown to regulate tumorigenicity and invasiveness, and accelerate metastasis [11–16]. Numerous prior studies have demonstrated that CSF1R signaling in TAMs promotes a protumorigenic and immunosuppressive M2-like phenotype. M2-polarized TAMs enhance tumor progression by contributing to angiogenesis, cancer cell invasion, metastasis, and T cell suppression through the secretion of vascular epithelial growth factors (VEGFs), IL10, programmed death-ligand 1 (PD-L1), and TGFβ [17]. In addition, M2 macrophages are known drivers of chemoresistance, furthering therapy failure in patients [18].

In recent years, several preclinical and clinical trials have explored the combination of CSF1/CSF1R inhibitors with various immunotherapies, such as immune checkpoint blockade, chemotherapy, and radiotherapy in several tumor models [19–29]. Although promising results were noted early in preclinical studies, clinical trials have reported limited anti-tumor responses. For instance, a recent randomized phase II study showed that the combination of the anti-CSF1 monoclonal antibody lacnotuzumab (MCS110) with gemcitabine and carboplatin yielded comparable anti-tumor efficacy to gem-carbo alone in a total of 50 patients with advanced triple-negative breast cancer (TNBC) [27]. Moreover, in another randomized phase II study, the combination of the anti-CSF1R monoclonal antibody cabiralizumab with anti-PD-1 (nivolumab) yielded limited activity in advanced pancreatic ductal adenocarcinoma (PDAC) [30]. So far, one CSF1R inhibitor (pexidartinib) has obtained FDA approval but only in the setting of tenosynovial giant cell tumors, a tumor type that aberrantly expresses CSF1 in neoplastic cells due to chromosomal translocations involving the CSF1 gene [31, 32].

Research has shown that cancer vaccines targeting tumor-associated antigens can further diversify immunity and mediate an effective anti-tumor immune response by enhancing the presence of tumor-reactive T cells in murine models of colon and mammary carcinoma [33]. When combined with additional immune mediators, the addition of an adenoviral-based neoepitope vaccine can increase T-cell receptor (TCR) diversity and promote epitope spreading, resulting in greater tumor control [34]. Currently, there is a gap in knowledge regarding the impact of combining inhibitors of the CSF1/CSF1R signaling axis with cancer vaccines and whether this approach could enhance the anti-tumor responses in the tumor microenvironment of solid tumors.

In this study, we developed and characterized CSF1 CRISPR-Cas9 knockouts in the 4T1 mammary and MC38 murine colon carcinoma cell lines to further understand the effects of CSF1 depletion in vitro and in vivo and determined its impact in tumor growth, tumor microenvironment, and epitope spreading. In addition, we evaluated the anti-tumor efficacy of combining CSF1 blockade with cancer vaccines in 4T1 and MC38 tumor models and further characterized its effect within the tumor microenvironment. This study demonstrates CSF1/CSF1R signaling inhibition enhances the expansion of neoepitope-specific T cells and promotes an immune-permissive tumor microenvironment. Overall, this study provides a more complete understanding of the potential benefit of antagonizing the CSF1/CSF1R pathway in combination with therapeutic cancer vaccines in breast and colon carcinoma models.

## Material and methods

### Cell culture

The MC38 cell line was propagated in RPMI1640 with l-glutamine (Corning) supplemented with 10% (v/v) FBS (Atlanta Biologicals) and 1% (v/v) antibiotic/antimitotic solution (Corning). 4T1-pCMV cells transfected to carry the PCDNA3.1 + plasmid (ThermoFisher Scientific) were propagated in RPMI1640, 10% FBS, 1% antibiotic/antimitotic media supplemented with 250 ug/mL of G418 (ThermoFisher Scientific). All cells were cultured at 37 °C, 5%CO2.

### CSF1 CRISPR

CRISPR knockouts were generated by co-transfecting cells with recombinant Cas9 protein version 2 and one of two TrueGuide Synthetic guide RNAs targeting murine CSF1 (either GCCTTCTTTAGGTAGCAAAC or TGTAGCCACATGATTGGGAA) with the Lipofectamine CRISPRMAX transfection reagent (ThermoFisher Scientific) using the manufacturer’s recommended protocol. Cells were incubated with the transfection reagents for 48 h prior to single-cell sorting into 96-well plates. Screening was performed by M-CSF1 ELISA (ThermoFisher Scientific) following expansion of single cell clones.

### Cytokine/chemokine assessment

ELISA assays of CSF1 were performed using the Invitrogen Mouse M-CSF1 (CSF1) ELISA kit (ThermoFisher Scientific) following the manufacturer’s instructions using either cell culture supernatants or mouse sera. IL-34 levels in mouse sera were assessed using the LegendMax Mouse IL-34 ELISA kit purchased from Biolegend.

The Proteome Profiler array Mouse XL Cytokine array kit (R&D Systems) was performed using cell culture supernatants. Briefly, 2 × 10^5^ cells per well were seeded on a 6-well plate. Following a 24-h incubation, the cells were washed with PBS, and media was replaced with RPMI serum-free media. Twenty-four h later, cell supernatant was collected and centrifuged at 500 g for 5 min to remove cell debris. The assay was run using the manufacturer’s recommended protocol and imaged using the LI-COR Odyssey Infrared Imaging System. Image analysis was performed using the ImageJ software.

### Cell proliferation

Cells were seeded into a 96-well white-walled, clear bottom plate (1000 cells/well in a volume of 100 μL), and growth was assessed at 24, 48, and 72 h using the Cell Titer Glo reagent (Promega). Luminescence was measured using an Envision microplate reader (PerkinElmer).

### Real-time PCR arrays

RNA was extracted from cells using the QIAgen RNAeasy Plus Mini Kit. Following DNAse I treatment (ThermoFisher Scientific), cDNA was generated using PrimeScript strand cDNA synthesis kit (Takara), and template mRNA was removed by treatment with RNAse H (Biolabs). Resulting cDNA was purified using the QIAquick PCR purification kit (Qiagen).

The RT^2^ Profiler PCR Array Mouse Epithelial to Mesenchymal Transition and the Mouse Chemokines and Receptors Panels (Qiagen) were used with 5 ng of purified cDNA as input, and the assay was run using a LightCycler96 real-time PCR instrument (Roche).

### Animals and tumor implantation

All animal procedures reported in this study that were performed by NCI-CCR affiliated staff were approved by the NCI Animal Care and Use Committee (ACUC) in accordance with federal regulatory requirements and standards. All components of the intramural NIH ACUC program are accredited by AAALAC International. C57BL/6 and BALB/c mice were bred and housed at the NIH. All studies utilized female C57BL/6 or BALB/c animals. Tumors were induced by implanting 3 × 10^5^ MC38 tumor cells subcutaneously or 5 × 10^4^ 4T1-pCMV tumor cells subcutaneously into the mammary fat pad of animals.

### Cell isolation and preparation

Spleens were harvested in LPA Media (without BME), dissociated through 70-mm filters, and subjected to ACK lysis (Quality Biological) to obtain splenocytes for analysis. Tumors were harvested in RPMI with 5% FBS media, cut into small pieces, and incubated for 30 min at 37ºC and 300 rpm in a digestion cocktail composed of RPMI supplemented with 5% (v/v) FBS, 2 mg/mL Collagenase Type I and IV (Worthington Biochemical Corporation), and 40 U/mL DNase I (Calbiochem). Following digestion, tumors were ground through 70-mm filters, spun for 5 min at 500 g, and resuspended in media for cell counting and subsequent flow cytometry analysis. Mice serum was collected into serum tubes with separating gel (BD BioSciences), rested for 30 min, and centrifuged at 2000 rpm for 2 min. Supernatant was stored at − 20 ºC until analysis.

Lungs were collected, rinsed with PBS 1 × , and weighed. They were then transferred to gentleMACS M tubes (Miltenyi Biotec) and the appropriate amount of PBS 1X was added based on weight (80 μL of PBS 1 × for every 25 mg of tissue). The tissue was mechanically disrupted using the gentleMacs dissociator (Miltenyi Biotec), and 80 μL aliquots of tissue homogenate were stored in DNAse/RNAse free tubes at  − 80 ºC until further processing.

### Real-time PCR-based lung metastasis assay

Lung tissue homogenate aliquots were thawed, and DNA extraction was performed using the QIAamp DNA Mini Kit (Qiagen). Upon addition of lysis buffer, samples were spiked with 10 μL of the TaqMan™ Universal DNA Spike-In Control (ThermoFisher Scientific), which was used as an internal standard. A real-time PCR assay was set up using 1 μL of neomycin resistance gene probe (Forward primer: CTCCTGCCGAGAAAGTATCCA; reverse primer: GCCGGATCAAGCGTATGC; Probe: CGCCGCATTGCATCAGCCAT; ThermoFisher Scientific), 1 μL of TaqMan Assay for the XenoTM DNA control (ThermoFisher Scientific), 8 μL of DNA template, 10 μL of TaqMan Fast Advanced master mix (ThermoFisher Scientific), and an appropriate amount of PCR-grade water for a total volume of 20 μL per reaction. The real-time PCR assay was run using a LightCycler96 real-time PCR instrument (Roche). Delta CT (*Δ* CT) was determined as the CT of neomycin expression minus the CT of the DNA spike control; hence, relative expression was calculated as 2^−*Δ*CT^. A standard curve was prepared using lungs from tumor-less mice spiked with different amounts of 4T1-pCMV cells for quantification purposes (Supplementary Fig. 1).

### Flow cytometric assays

All antibodies used for flow cytometric analysis are described in Supplementary Table 1. Cell populations were identified as follows: CD8 + T cells: live/CD45 + CD3 + CD8 + ; CD4 + T cells: live/CD45 + CD3 + CD4 + FoxP3 − ; Central memory: CD44 + CD62L + ; Effector memory: CD44 + CD62L − ; T regulatory: live/CD3 + CD45 + CD4 + FoxP3 + ; M1 macrophages: live/CD45 + CD11b + F4/80 + CD38 + ; M2 macrophages: live/CD45 + CD11b + F4/80 + CD38-CD206 + ; Mono myeloid-derived suppressive cells (MDSC): live/CD45 + CD11b + F4/80-Ly6C + Ly6G-; Gran MDSC: live/CD45 + CD11b + F4/80-Ly6C-Ly6G + ; Macrophages: live/CD45 + /CD11b + /F4/80 + . Data were collected using an Attune NxT flow cytometer and analyzed using FlowJo (v10, BD Biosciences).

### Vaccination and treatments

Animals were vaccinated with MC38 neoepitope vaccine, as previously reported [34]. Briefly, mice were vaccinated with a pool of four peptides (100 μg each peptide) consisting of Ptgfr, Trp53, Olfr66, and Jak1 (GenScript). The peptides were diluted in PBS 1×, emulsified in Montanide ISA 51 VG (Seppic), and administered subcutaneously. Murine anti-CSF1R was kindly provided by Syndax as part of Collaborative Research and Development Agreement (CRADA) with the National Cancer Institute. Mice were treated with 500 μg (i.p) of anti-CSF1R antibody diluted in PBS at days 10, 12, and 14 following tumor implantation. Anti-PD-L1 (10F.9G2, BioXCell, 200 μg) was administered intraperitoneally and diluted in PBS. Adeno-TWIST1 vaccine (Vector Biolabs, 10^10^ viral particles) was also diluted in PBS and injected subcutaneously.

### ELISPOT assays

Splenocytes or PBMCs were harvested, and ex vivo antigen-dependent cytokine secretion was assessed using IFNγ (BD Biosciences). ELISPOT assays were performed according to the manufacturer’s instructions. Target peptides (final concentration: 1 μg/peptide/mL) were incubated with 0.5–1.0 × 10^6^ splenocytes overnight. MC38 peptide pools used are described in Supplementary Table 2. Peptides used for stimulation in the 4T1 tumor model were previously identified by Kreiter et al. [35] and are described in Supplementary Table 3. ELISPOT data are adjusted to the number of spots/million splenocytes after subtracting the number of spots in paired wells containing a negative control peptide.

### Immunohistochemistry (IHC)

Tumors, fixed in Z-fix (Anatech), were paraffin-embedded and sectioned. Slides were stained for CD8 (Thermo; Clone: 4SM16), F4/80 (BioRad, Clone Cl:A3-1), and CD4 (Thermo; Clone: 4SM95) using the Opal Multiplex Immunohistochemistry Kits (PerkinElmer). Images were acquired using an Axio Scan.Z1 Slide Scanner (Zeiss).

### Network analysis by ingenuity pathway analysis (IPA)

The list of differentially expressed genes in the CSF1−/− 4T1 and MC38 cell lines, containing gene identifiers and corresponding expression values, was uploaded into the Ingenuity Pathway Analysis software (Qiagen), a manually curated database of functional interactions. The “core analysis” function included in the software was used to interpret the differentially expressed data, which included biological processes, canonical pathways, upstream transcriptional regulators, and gene networks. Results with an overlap *p* value < 0.05 were considered as differentially statistically significant.

### Statistical analysis

Statistical analyses were performed using GraphPad Prism (v9; GraphPad Software).

All data points represent the mean ± SEM and a *p* value < 0.05 was considered significant.

## Results

### CFS1 deletion promotes tumor control in the 4T1 and MC38 tumor models

**Fig. 1:**
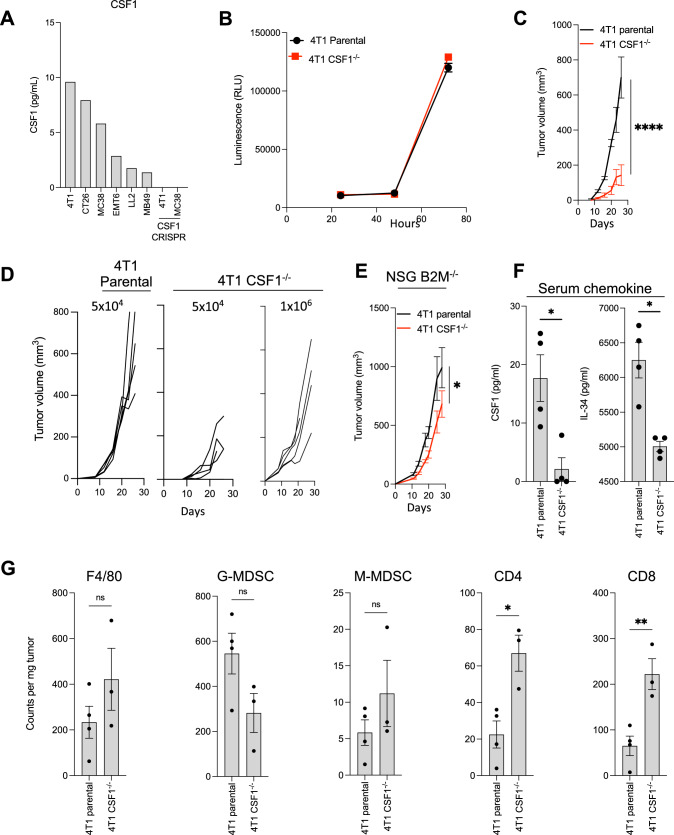
CFS1 deletion promotes tumor control in the “cold” 4T1 tumor model. **a** In vitro CSF1 production by parental and CRISPR knockout cell lines. **b** In vitro cell proliferation of 4T1 parental and CSF1^−/−^ cell lines (*n* = 12 replicates). **c** In vivo tumor growth of 4T1 parental and CSF1^−/−^ cell lines in BALB/c mice (*n* = 4–5 mice per group). **d** Tumor growth parental and two doses of CSF1^−/−^ 4T1 cells implanted in BALB/c mice (*n* = 5 mice per group). **e** In vivo tumor growth of 4T1 parental and CSF1^−/−^ cell in NSG B2m^−/−^ mice (*n* = 10 mice per group). **f** Serum CSF1 and IL-34 levels in BALB/c mice bearing day 26 4T1 parental or CSF1^−/−^ 4T1 tumors (*n* = 4–5 mice per group). **g** Flow cytometry analysis of immune cells infiltrating day 26 4T1 parental and CSF1^−/−^ tumors (*n* = 3–4 mice per group). Data shown as mean ± SEM. *p value ≤ 0.05, **p value ≤ 0.01, ****p value ≤ 0.0001

We first screened a panel of murine carcinoma cell lines to assess the production of tumor-CSF1 in vitro. Following analysis by ELISA, we observed that 4T1 and MC38 carcinoma cell lines produced high and intermediate amounts of CSF1, respectively (Fig. 1a). To assess the role of tumor-derived CSF1, we developed CRISPR-Cas9 knockouts in both the 4T1 mammary and MC38 colon carcinoma cell lines (Fig. 1a). Although deletion of CSF1 did not change the rate of 4T1 cell proliferation in vitro (Fig. 1b), we observed a dramatic decrease in the rate of tumor growth in BALB/c mice as compared to the 4T1 parental cell line (Fig. 1c). Similar tumor growth curves to the parental cell line were obtained only when implanting 20 times more CSF1−/− cells as compared to the parental cell line (Fig. 1d). Although the relative rate of growth of the knockout cells increased when implanted in immune-compromised animals, we continued to observe a significantly reduced rate of tumor growth as compared to the parental 4T1 cell line (Fig. 1e). Animals bearing CSF1−/− tumors also showed decreased levels of serum CSF1 as compared to the parental 4T1 tumors, suggesting tumor cells are a significant source of CSF1 present in tumor-bearing animals (Fig. 1f).

We next harvested tumors from animals bearing either 4T1 parental or CSF1−/− tumors to assess the impact of CSF1 knockout within the tumor microenvironment. Flow.cytometric analysis revealed a significant increase in the absolute count of CD4 T cells and CD8 T cells when comparing CSF1−/− tumors to the parental cell line. Surprisingly, no major differences were noted in TAMs (F4/80) and MDSC (Fig. 1g). This lack of impact on the monocytes in our knockout tumor may be due to a coordinated increase in Ccl2 production by the knockout cell line, which is known to recruit monocytes to the tumor microenvironment (Supplementary Fig. 2a).

**Fig. 2:**
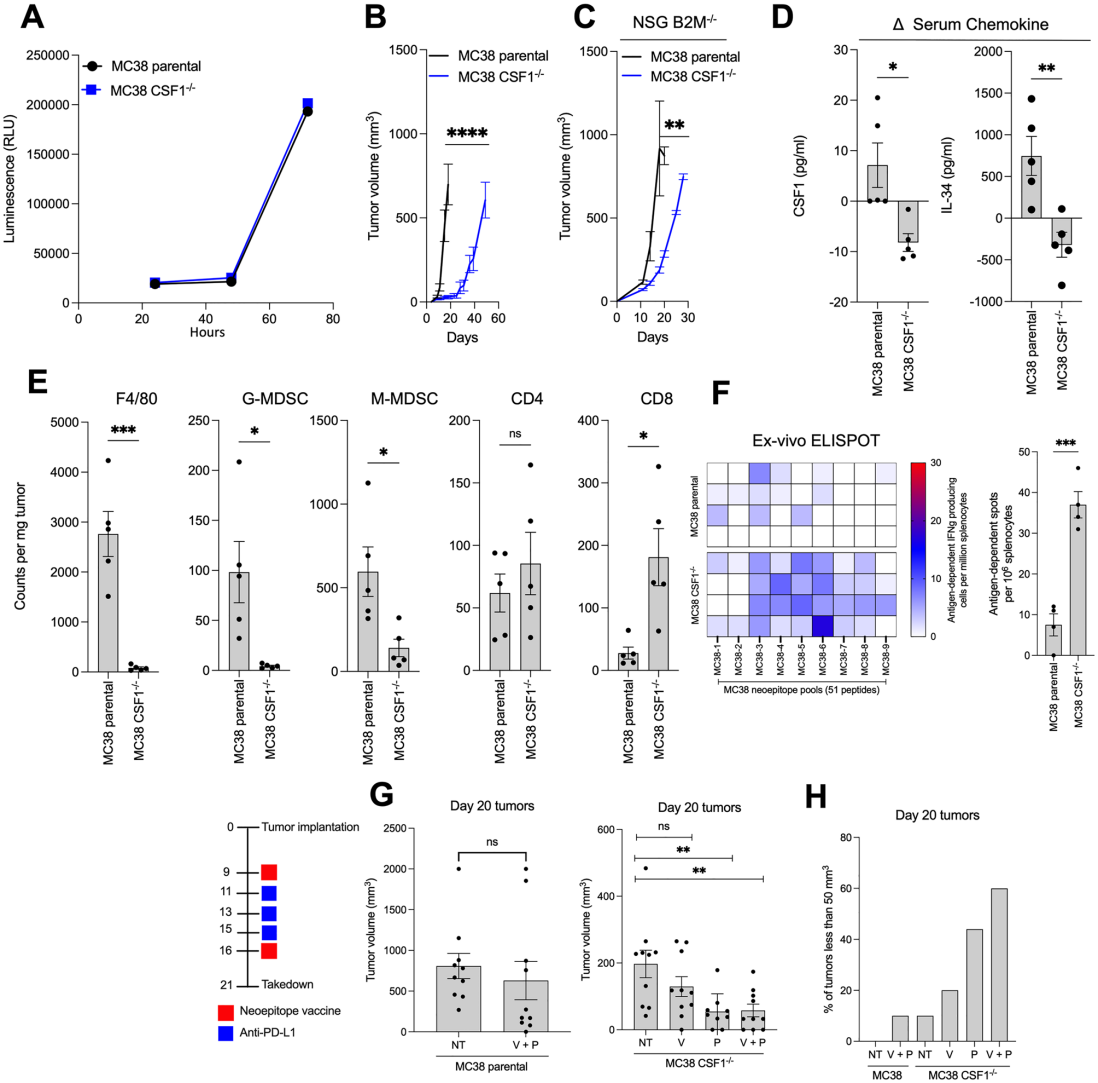
Deletion of CSF1 enhances tumor growth inhibition in the MC38 tumor model. **a** In vitro cell proliferation of MC38 parental and CSF1^−/−^ cell lines (*n* = 12 replicates). **b** In vivo tumor growth of MC38 parental and CSF1^−/−^ cell lines in C57BL6 mice (*n* = 5 mice per group). **c** In vivo tumor growth of MC38 parental and CSF1^−/−^ cell lines in NSG B2m^−/−^ mice (*n* = 5 mice per group). **d** CSF1 and IL-34 serum level changes as compared to baseline levels in mice bearing day 32 tumors (*n* = 5 mice per group). **e** Flow cytometry analysis of immune cells infiltrating day 23 MC38 parental and CSF1^−/−^ tumors (*n* = 4–5 mice per group). **f** IFNy ELISPOT analysis by splenocytes on day 21 of tumor growth against neoepitopes identified in the MC38 model. Each row represents one mouse (*n* = 4 mice per group). **g** Day 20 tumor volumes of mice treated as indicated (*n* = 10 mice per group). **h** Percentage of tumors less than 50 mm^3^ following treatments with either neoepitope vaccine, anti-PD-L1 or combination therapy in MC38 parental and MC38 CSF1 knockout tumors. Data shown as mean ± SEM. *p value ≤ 0.05, **p value ≤ 0.01, ***p value ≤ 0.001, ****p value ≤ 0.0001

Next, we evaluated the anti-tumor efficacy of CSF1 deletion in the MC38 tumor model. Knocking out CSF1 expression in the MC38 model did not affect the ability of these cells to proliferate in vitro as compared to the parental cell line (Fig. 2a); however, we did observe a dramatic reduction in the rate of growth by CSF1−/− tumors, as compared to the parental line in C57BL/6 mice (Fig. 2b). This reduced rate of tumor growth was less evident when tumors were implanted in immune-compromised animals (Fig. 2c). Similar to our observations in the 4T1 model, lower levels of CSF1 were detected in the serum of mice bearing MC38 CSF1 knockouts as compared to parental tumors (Fig. 2d). Further evaluation of the MC38 CSF1−/− cell line by RT-PCR revealed a marked increase in Ccl20 expression, accompanied by downregulation of chemokines Cxcl3 and Ccl11, and several genes associated with the epithelial to mesenchymal transition (EMT) process (Supplementary Fig. 2C).

We next interrogated the impact of CSF1 deletion in the MC38 tumor microenvironment. CSF1−/− tumors displayed decreased infiltration in TAMs and MDSCs (Fig. 2e). These effects were paralleled with significant elevations in CD8 T cells in CSF1−/− MC38 tumors when compared to the parental. Moreover, the deletion of CSF1 also promoted the expansion of immunity generated against MC38 neoepitopes in tumor-bearing mice (Fig. 2f).

Following our observations of enhanced epitope spreading in animals bearing MC38 CSF1−/− tumors, we asked whether a neoepitope targeting vaccine would further improve anti-tumor outcomes. Mice bearing MC38 parental or MC38 CSF1 knockout tumors were treated with neoepitope vaccine, anti-PD-L1, or combination therapy. Whereas the anti-tumor effect in MC38 parental tumors was limited, we observed significant tumor growth inhibition in MC38 CSF1 knockout tumors following treatment with immune checkpoint blockade alone or in combination with neoepitope vaccine (Fig. 2g, Supplementary Fig. 3a–c). Combination therapy resulted in the greatest degree of tumor control in the MC38 CSF1−/− tumor model, with more than half of the mice bearing smaller tumors when compared to the other groups at the end of the study (Fig. 2h).

### IPA of CSF1−/− 4T1 and MC38 tumor models

To further understand the potential tumor cell-centric mechanisms resulting from knocking out CSF1 expression in both the 4T1 and MC38 tumor models, we assessed the expression of 178 genes by real-time-PCR array (Supplementary Fig. 2). We observed that the top canonical pathways for both 4T1 and MC38 CSF1 knockout cell lines were granulocyte adhesion and diapedesis (*p*-values = 7.94E − 19 and 1.58E − 21 for 4T1 and MC38, respectively) followed by agranulocyte adhesion and diapedesis [*p*-values = 3.16E − 16 (4T1 CSF1−/−) and 6.31E-19 (MC38 CSF1−/−)]. Other significant canonical pathways shared by both cell lines included the tumor microenvironment pathway and pulmonary fibrosis idiopathic signaling pathway, which were predicted to be inhibited in the MC38 CSF1 knockout cell line (*z*-scores =  − 1.9 and − 3.2, respectively). The 4T1 CSF1−/− cell line also overlapped with other pathways, such as the HOTAIR regulatory pathway (*p*-value = 2.51E − 08) and the differential regulation of cytokine production in macrophages and T helper cells by IL-17a and IL-17F pathway (*p*-value = 1.07E−06) (Table 1).

**Table 1:** Top canonical pathways in 4T1 and MC38 CSF1 −/− cell lines

Model	Ingenuity canonical pathways	*P*-Value	Molecules
4T1 CSF1 −/−	Granulocyte adhesion and diapedesis	7.94 E− 19	ACKR3, Ccl2, CCL25, CCL5, Ccl7, CCR10, Cxcl3, CXCR3, CXCR4, FPR1, MMP3, MMP9
	Agranulocyte adhesion and diapedesis	3.16 E− 16	ACKR3, Ccl2, CCL25, CCL5, Ccl7, CCR10, Cxcl3, CXCR3, CXCR4, MMP3, MMP9
	Pulmonary healing signaling pathway	2.04 E− 09	ACKR3, CDH1, CXCR4, MMP3, MMP9, TGFB2, WNT11
	HOTAIR regulatory pathway	2.51 E− 08	CDH1, ESR1, MMP3, MMP9, SPP1, WNT11
	Tumor microenvironment pathway	4.37 E− 08	CXCR4, IL6, MMP3, MMP9, SPP1, TGFB2
	Pulmonary fibrosis idiopathic signaling pathway	6.17 E− 08	CDH1, IL17A, IL6, MMP3, MMP9, TGFB2, WNT11
	Role of osteoblasts, osteoclasts and chondrocytes in rheumatoid arthritis	1.66 E− 07	BMP7, IL17A, IL6, MMP3, SPP1, WNT11
	FAT10 cancer signaling pathway	2.95 E− 07	ACKR3, CXCR4, IL6, TGFB2
	Colorectal cancer metastasis signaling	5.13 E− 07	CDH1, IL6, MMP3, MMP9, TGFB2, WNT11
	Differential regulation of cytokine production in macrophages and T helper cells by IL-17A and IL-17F	1.07 E− 06	CCL5, IL17A, IL6
MC38 CSF1 −/−	Granulocyte adhesion and diapedesis	1.58 E− 21	CCL1, CCL11, CCL2, CCL20, Ccl6, Ccl7, CCR9, CXCL10, Cxcl11, CXCL16, Cxcl3, Cxcl9, FPR1, ITGB2, Ppbp, TNFRSF11B
	Agranulocyte adhesion and diapedesis	6.31 E− 19	CCL1, CCL11, CCL2, CCL20, Ccl6, Ccl7, CCR9, CXCL10, Cxcl11, CXCL16, Cxcl3, Cxcl9, FN1, ITGB2, Ppbp
	Pulmonary fibrosis idiopathic signaling pathway	1.00 E− 14	CDH2, COL1A2, COL3A1, COL5A2, EGFR, FN1, FZD7, IL6, JAG1, SNAI1, STAT3, TCF7L1, TGFB2, TGFB3
	Regulation of the epithelial mesenchymal transition by growth factors pathway	1.00 E− 14	CDH2, EGFR, FGF21, IL6, OCLN, SNAI1, STAT3, TGFB2, TGFB3, TNFRSF11B, ZEB1, ZEB2
	Hepatic fibrosis/hepatic stellate cell activation	1.26 E− 14	CCL2, COL1A2, COL3A1, COL5A2, CSF1, EGFR, FN1, IGFBP4, IL6, TGFB2, TGFB3, TNFRSF11B
	Regulation of the epithelial–mesenchymal transition pathway	1.26 E− 14	CDH2, EGFR, FGF21, FZD7, JAG1, SNAI1, STAT3, TCF7L1, TGFB2, TGFB3, ZEB1, ZEB2
	Tumor microenvironment pathway	2.00 E− 13	CCL2, COL1A2, COL3A1, CSF1, FGF21, FN1, IL6, SPP1, STAT3, TGFB2, TGFB3
	Hepatic fibrosis signaling pathway	1.10 E− 10	CCL2, COL1A2, COL3A1, FZD7, ITGB2, SNAI1, SPP1, STAT3, TCF7L1, TGFB2, TGFB3, TNFRSF11B
	Pulmonary healing signaling pathway	5.50 E− 10	EGFR, FZD7, JAG1, STAT3, TCF7L1, TGFB2, TGFB3, TLR2, TNFRSF11B
	Wound healing signaling pathway	4.47 E− 09	COL1A2, COL3A1, COL5A2, EGFR, FN1, IL6, TGFB2, TGFB3, TNFRSF11B

Additionally, the MC38 CSF1−/− cell line showed significant overlap with the regulation of EMT by growth factors (*p*-value = 1.00E − 14, *z*-score =  − 2.7) and wound healing signaling canonical pathways (*p*-value = 4.47E − 09, z-*s*core =  − 1.7). Pro-tumorigenic processes such as cell movement, colony formation, invasion of tumor cell lines, EMT, and migration of tumor cell lines were predicted to be inhibited. Upstream regulators TGF beta1 (*z*-score =  − 3.113), EGFR (*z*-score =  − 2.279), Ccl2 (*z*-score = –2.4) and CSF1 (*z*-score =  − 2.13) were also predicted to be inhibited. Overall, this data highlights the significance of CSF1 as a key regulator of the tumor microenvironment and as a promoter of the EMT process.

### Anti-CSF1R inhibition promotes an immune-permissive tumor microenvironment and amplifies immunity generated against neoepitopes

**Fig. 3:**
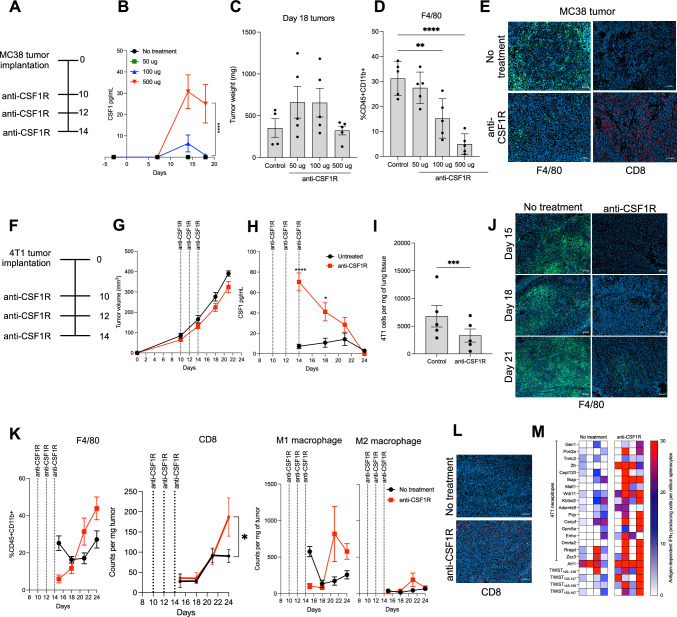
Anti-CSF1R inhibition promotes an immune-permissive tumor microenvironment and amplifies immunity generated against neoepitopes in tumor-bearing mice. **a** Timeline of anti-CSF1R antibody administration. **b** Serum CSF1 levels in mice treated with 50, 100 or 500 µg of CSF1R blocking antibodies in MC38 tumor-bearing mice (*n* = 4–5 mice per group). **c** Weight of day 18 MC38 tumor harvested from animals treated with indicated amounts of CSF1R antibody. **d** Abundance of tumor-associated macrophages in day 18 MC38 tumors harvested from animals treated as indicated (*n* = 5 mice per group). **e** Representative immunofluorescent images of F4/80 + cells (green signal) and CD8 (red signal) in day 18 tumors harvested from either control animals or animals treated with three 500 ug doses of CSF1R blocking antibody. Blue signal corresponds to DAPI staining. Scale bar, 100 μm. **f** Timeline of the administration of 500 ug doses of CSF1R antibody and tissue collection following 4T1 tumor implantation (*n* = 3–5 mice per timepoint). **g** In vivo tumor growth of 4T1 tumors in BALB/c mice after treatment with anti-CSF1R (*n* = 14–20 mice per group). **h** Kinetics of serum CSF1 level changes following three administrations of 500 µg of CSF1R antibody in 4T1 tumor-bearing mice as determined by ELISA (*n* = 14–20 mice per group). **i** Enumeration of lung metastasis of 4T1 cells at day 21 post-tumor implantation after anti-CSF1R treatment (*n* = 5 mice per group). **j** Representative immunofluorescent images of F4/80 + cells (green signal) in tumors collected on days 15, 18 and 21 of tumor growth from either control animals or animals treated with three 500 ug doses of CSF1R blocking antibody. Blue signal corresponds to DAPI staining. Scale bar, 100 μm. **k** Kinetic changes of tumor-infiltrating F4/80 + , CD8 + , M1 and M2 macrophages from either control animals or animals treated with three 500 µg doses of CSF1R blocking antibody. **l** Representative immunofluorescent images of CD8 + cells in day 21 tumors harvested from either control animals or animals treated with three 500 µg doses of CSF1R blocking antibody. Blue signal corresponds to DAPI staining. Scale bar, 100 μm. **m** IFNy ELISPOT analysis of mice bearing 4T1 tumors following treatment with three doses of 500 µg CSF1R antibody. Splenocytes were harvested and incubated with 4T1 neoepitopes, and three TWIST1 25-mer peptides. Each column represents one mouse (*n* = 4 mice per group). Data shown as mean ± SEM. *p value ≤ 0.05, **p value ≤ 0.01, ****p value ≤ 0.0001

We next sought to assess the impact of an antagonistic CSF1R antibody when administered to mice bearing either MC38 or 4T1 tumors. Utilizing the treatment regimen outlined in Fig. 3a, we observed a sharp increase in CSF1 serum levels following treatment with 500 ug of anti-CSF1R in the MC38 tumor model, which is characteristic of receptor occupancy by the antibody (Fig. 3b). Although no differences in tumor weight were observed in treated animals (Fig. 3c), a decrease in TAMs was seen following treatment (Fig. 3d, e left panel). Moreover, increased infiltration of CD8 T cells into tumors was also noted by immunohistochemistry (IHC) (Fig. 3e right panel).

Using the treatment regimen outlined in Fig. 3f, we did not notice any impact of CSF1 receptor blockade on the growth of 4T1 tumors (Fig. 3g). Similar to our observations in the MC38 model, there was a rapid increase in serum CSF1 in treated animals, which declined gradually over time following treatment completion (Fig. 3h). Whereas we did not observe any impact on the growth of the primary tumor, a reduction in the rate of metastasis was noted in treated animals (Fig. 3i). A decrease in TAMs was also seen in the mice that received the anti-CSF1R, which persisted until at least one week following treatment as assessed by IHC (Fig. 3j). Nonetheless, using a flow cytometric-based assay, TAMs subsequently recovered 10 days after treatment completion. We also observed an increase in CD8 T cells and a trend for an increase in M1 macrophages in the tumor following the cessation of treatment (Fig. 3k). Further analysis by IHC in tumors confirmed the increase in CD8 T cell infiltration after anti-CSF1R treatment (Fig. 3l). To further dissect the immune responses to CSF1R inhibition, we performed ELISPOT assays to examine the impact of anti-CSF1R treatment in epitope spreading in mice bearing 4T1 tumors. Interestingly, CSF1R inhibition enhanced immunity generated against TWIST1 and several TAMs expressed by the 4T1 model (Fig. 3m, Supplementary Fig. 4).

**Fig. 4:**
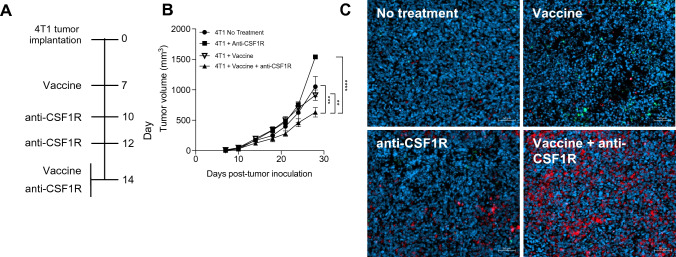
Combination therapy using vaccine and CSF1 signaling inhibition enhances CD8 T cell infiltration in tumor-bearing mice**. a** Timeline of CSF1R antibody (500 µg per timepoint) and administration of the adeno-TWIST1 vaccine treatment following 4T1 tumor implantation. **b** In vivo tumor growth of 4T1 tumors in BALB/c mice after treatment with anti-CSF1R and adeno-TWIST1 vaccine (*n* = 3–7 mice per group). **c** Representative immunofluorescent images of CD4 cells (green signal) and CD8 (red signal) CD8 + cells in day 21 tumors harvested from animals from indicated treatment groups. Blue signal corresponds to DAPI staining. Scale bar, 50 μm. Data shown as mean ± SEM. *p value ≤ 0.05, **p value ≤ 0.01

Following our observations of epitope spreading and modulation of the M1/M2 phenotype post-CSF1R blockade, we asked whether the combination of anti-CSF1R therapy with an adeno-TWIST1 vaccine would result in improved tumor control. Using the treatment regimen outlined in Fig. 4a, we found that the combination of anti-CSF1R and vaccine elicited significant tumor control compared to the untreated, anti-CSF1R monotherapy and vaccine monotherapy groups (Fig. 4b). Moreover, combination therapy also resulted in a synergistic increase in CD8 T cell infiltration in tumors (Fig. 4c). The observed increase in CD8 infiltration in the combination therapy group was greater than the one observed with anti-CSF1R and vaccine monotherapy groups. Hence, these findings suggest that the combination of CSF1/CSF1R signaling blockade with vaccine promoted an immune-permissive tumor microenvironment in the 4T1 tumor model.

## Discussion

CSF1 is a major regulator of TAM recruitment to the tumor microenvironment that has been linked to poor prognosis and increased tumor invasiveness in several cancer types. Our study used a multiapproach perspective to understand further the immune effects, anti-tumor responses, and molecular mechanisms that might be involved in CSF1 blockade in murine breast and colon tumor models. Here, we assessed the impact of tumor-derived CSF1 by developing CSF1 CRISPR-Cas9 knockouts in the 4T1 mammary carcinoma and MC38 murine colon carcinoma cell lines, where mice harboring these tumors achieved tumor control when compared to the parental tumors. Overall, we demonstrated that CSF1/CSF1R signaling inhibition promotes an immune-permissive tumor microenvironment and enhances neoepitope spreading, resulting in enhanced tumor control.

Prior research focusing on the targeting of the CSF1/CSF1R signaling axis had shown varied results, from promising data in preclinical models [29] to limited anti-tumor effects later in clinical trials [36]. For example, previous studies highlighted the potential benefits of combining CSF1R inhibitors with immune checkpoint blockade preclinically; however, most clinical trials testing this combination have yielded insufficient anti-tumor efficacy [19–26]. In a phase Ib/II study, of a total of 116 patients bearing advanced solid tumors who received an anti-colony-stimulating factor 1 receptor antibody (AMG 820) in combination with pembrolizumab, only three patients obtained immune-related partial responses [23]. Research has shown that vaccines targeting neoepitopes can boost the response to checkpoint blockade by enhancing the presence of tumor-reactive T cells [33, 34]. Currently, few studies have investigated the combination of cancer vaccines with CSF1/CSF1R inhibition. In a study by Saung et al., the administration of anti-CSF1R therapy before and after the combination of a GM-CSF-secreting pancreatic cancer vaccine with anti-PD1 resulted in increased survival and higher intratumoral infiltration of CD8 and CD4 T cells [37]. Here, the combination of neoepitope vaccine with anti-CSF1R resulted in a synergistic increased tumor infiltration of CD8 T cells accompanied by epitope spreading and decreased tumor growth. The increased presence of CD8 T cells is significant in this regard, since it has been previously shown that the added benefit of combination with CSF1R targeting agents is lost when specifically depleting CD8 Tcells [38, 39]. Hence, our findings further provide a rationale for combining cancer vaccines with CSF1 targeting agents in the future.

Further characterization of our CSF1 knockout cell lines revealed several factors that could have contributed to the observed anti-tumor effect following vaccination. In the MC38 CSF1−/− model, we observed increased CCL20 (MIP-3α) and CCR9 mRNA expression. CCL20 is an inflammatory chemokine previously linked to dendritic cell (DC) migration. A previous study found that combining tumor cells expressing CCL20 followed by DC vaccination yielded strong anti-tumor responses [40]. On the other hand, CCR9 is the unique receptor of CCL25, a chemokine whose expression increased in our 4T1 CSF1−/− tumor cell line. Interestingly, previous studies have shown that this chemokine is not expressed in human or murine TNBC and that intratumoral delivery of CCL25 enhances anti-tumor effects by stimulating CCR9 + CD8 + T cell tumor infiltration [41]. We also found increases in CCL2, CCL7, and CCL5 expression in our 4T1 CSF1−/− model, which are inflammatory chemokines known to guide immature DC migration to inflammation sites and become activated. CCL5 has previously been tested in murine models as adjuvant therapy for tumor lysate-pulsed DC vaccines [42]. Further studies are needed to validate the chemokine composition within the tumor microenvironment following combination therapy of CSF1R targeting agents and cancer vaccines. In addition, the 4T1 tumor model is known to be heavily infiltrated with MDSCs, which are known to suppress T cell activity and dampen antitumor immunity [43]. The development of MDSCs appears to be modulated by the same growth factors that regulate normal myelopoiesis, such as GM-CSF, G-CSF, and CSF1 [44]. Previous studies have demonstrated that MDSCs decrease the activation of antigen-presenting cells, natural killer (NK) and T cells in the TME resulting in immunosuppression and tumor progression [45]. Hence, targeting the CSF1/CSF1R could potentially decrease MDSC expansion and activity in the 4T1 tumor model, resulting in enhanced efficacy of therapeutic cancer vaccines.

Previous studies have demonstrated highly heterogeneous responses following treatment with CSF1 receptor inhibitors in the tumor microenvironment across different tumor models [46]. In a study performed with a murine model of hepatocellular carcinoma, treatment with a CSF1R inhibitor resulted in increased CD8 infiltration accompanied by a reduction in MDSCs, a profile similar to what we observed in our study with the MC38 CSF1 knockout model [47]. In contrast, other studies have reported different effects, such as increased recruitment of MDSCs, increased frequency of neutrophils, and decreased infiltration of CD4 and Tregs in various tumor models [9, 48]. A study by Ries et al. showed significant increases in CD4, CD8, and NK cells with no significant changes in Tregs following treatment with an anti-CSF1R antibody in the MC38 model [29]. These results further highlight the immune heterogeneity in the tumor microenvironment following CSF1/CSF1R signaling inhibition in different tumor models. In our study, both 4T1 and MC38 models showed increased CD8 T cell infiltration in tumors and a reduction of TAMs following blockade with an anti-CSF1R antibody. Nonetheless, some differences in myeloid composition were noted. Previous studies have highlighted the differences between these two tumor model systems [49–51]. MC38 tumors are known to have a higher tumor mutational burden as compared to 4T1 tumors [49]. In addition, MC38 colon tumors have been described as immunologically “warm,” with a higher degree of T cell infiltration, dendritic cell infiltration, and response to immune checkpoint blockade [51]. On the other hand, the 4T1 mammary tumor model is an immunologically “cold” preclinical model, which is refractory to PD-L1 blockade. These tumors are poorly infiltrated with T cells and there is an abundance of immunosuppressive cells such as MDSCs within the tumor microenvironment [38, 49, 50]. Hence, we believe that these characteristics may contribute to the differences in myeloid composition observed in the 4T1 and MC38 models in our study. It remains to be established whether other factors, such as collaborative interactions between tumor macrophages and stromal cells, including cancer-associated fibroblasts (CAFs), might play a key role in regulating the immune responses to CSF1/CSF1R blockade in the tumor microenvironment in these models.

Another interesting finding was that both of our CSF1 knockout models downregulated the expression of various genes associated with the EMT process (Supplementary Fig. 2), including SPP1. SPP1 is a secreted chemokine-like glycophosphoprotein linked to pro-tumorigenic processes such as tumor cell invasion, proliferation, and metastasis [52]. Higher SPP1 expression has previously been associated with poor prognosis in breast and pancreatic cancer [53, 54]. Further studies need to be conducted to understand better the mechanism by which CSF1 and SPP1 expression are associated.

In future studies, it will be important to further explore the mechanisms by which the combination therapy of CSF1 inhibition with vaccine drives tumor suppression within the tumor microenvironment, including the precise contribution of DC subsets. In addition, it remains to be investigated whether combinations involving pro-inflammatory cytokines would further enhance anti-tumor efficacy. For example, the addition of IL-12 tumor-targeted antibodies to the combination could further mediate tumor suppression responses by promoting the activation of T lymphocytes, NKs, and antigen-presenting cells while enhancing IFNy secretion [55].

To conclude, our findings highlight the therapeutic potential of combining CSF1/CSF1R pathway targeting agents with neoepitope targeting vaccines to further modulate anti-tumor immune responses in the tumor microenvironment. Our study provides a greater understanding of the combination of CSF1 inhibitors with neoepitope vaccines in breast and colon cancer tumor models while also providing greater insight into the molecular mechanisms and effects caused by intratumoral depletion of CSF1.

## Supplementary Information

Below is the link to the electronic supplementary material.

Supplementary file1 (DOCX 336 KB)

## Abbreviations

CAF: Cancer-associated fibroblast
CSF1: Colony-stimulating factor 1
M-CSF: Macrophage colony-stimulating factor
MDSC: Myeloid-derived suppressive cell
TAM: Tumor-associated macrophage
VEGF: Vascular epithelial growth factor
