# Supplementary material for: Blockade of tumor-derived colony-stimulating factor 1 (CSF1) promotes an immune-permissive tumor microenvironment
Source: Cancer Immunol Immunother. 2023 Jul 28;72(10):3349–62. doi: 10.1007/s00262-023-03496-2 (PMC10491706; doi:10.1007/s00262-023-03496-2)
Supplement: Supplementary file 1 — Supplementary file1 (DOCX 336 KB) [file 262_2023_3496_MOESM1_ESM.docx]

**Supplementary Table 1**: List of flow cytometry antibodies used in this study.

| **Target** | **Clone** | **Vendor** |
| --- | --- | --- |
| Ly6G | 1A8 | BioLegend |
| Ly6C | HK1.4 | ThermoFisher Scientific |
| CD274 (PD-L1) | MIH5 | ThermoFisher Scientific |
| CD45 | 30-F11 | ThermoFisher Scientific |
| iNOS | CXNFT | ThermoFisher Scientific |
| CD38 | 90 | BioLegend |
| CD115 | AFS98 | ThermoFisher Scientific |
| F4/80 | BM8 | BioLegend |
| CD11b | M1/70 | ThermoFisher Scientific |
| CD206 (MMR) | MR6F3 | ThermoFisher Scientific |
| Arg1 | A1exF5 | ThermoFisher Scientific |
| FoxP3 | 150D/E4 | ThermoFisher Scientific |
| CD44 | IM7 | Invitrogen |
| Ki67 | SolA15 | ThermoFisher Scientific |
| Granzyme B | NGZB | ThermoFisher Scientific |
| CD3e | 145-2C11 | BD Horizon |
| CD45 | 30-F11 | ThermoFisher Scientific |
| CD4 | RM4-5 | ThermoFisher Scientific |
| CD8 | 53-6.7 | ThermoFisher Scientific |
| CD62L | MEL-14 | ThermoFisher Scientific |

**Supplementary Table 2**: List of MC38 9-mer peptide library pools.

|  | **Gene** | **9-mer peptide sequence** |
| --- | --- | --- |
| MC38 peptide pool 1 | Adrbk1 | IVHGYMSKI |
|  | Aldh1l1 | TVVIKPAQL |
|  | Aoc2 | IVGHFYGGL |
|  | Carnmt1 | MMKYYYESV |
|  | Carnmt1 | MKYYYESVL |
|  | Cav2 | DAYSHHSGF |
| MC38 peptide pool 2 | Ccdc171 | TATRNDFTL |
|  | Ccdc51 | VTFLPTLPL |
|  | Ccdc51 | TCVTFLPTL |
|  | Cd180 | SLFQNLHLL |
|  | Cers1 | TSYRFFHDP |
|  | Cog1 | QLYLLCCQL |
| MC38 peptide pool 3 | Cpne1 | SSPYSLHYL |
|  | Cpne1 | YSLHYLSPT |
|  | Dnajc11 | NNEDYYSLL |
|  | Dpagt1 | ASIIVFNLL |
|  | Dvl1 | SSLTSSVPV |
|  | Entpd7 | SCRTFLSPL |
| MC38 peptide pool 4 | Flii | TSLECLSNL |
|  | Gtf2i | YVIPRLERI |
|  | Hacd2 | SYIPLFPHL |
|  | Hacd2 | LFPHLYFHM |
|  | Klhl2 | VLTRLRTPM |

|  | **Gene** | **9-mer peptide sequence** |
| --- | --- | --- |
| MC38 peptide pool 5 | Lrpprc | TAFQVLLPL |
|  | Mdn1 | LSRAFRTRF |
|  | Mdn1 | AFRTRFVEL |
|  | Med1 | FSLSFQHPV |
|  | Nfe2l2 | ASYSLVAHI |
|  | Npm1 | ISGQHLLAV |
| MC38 peptide pool 6 | Nsd1 | KGYRHKVPL |
|  | Ppm1b | IQLVNGSLA |
|  | Rbm17 | SAWEVLIPL |
| MC38 peptide pool 7 | Sart3 | SVTVFVNNL |
|  | Scaper | KVLQKLCQL |
|  | Slc35f6 | QILVFLILL |
|  | Spag5 | AQLQNLTST |
|  | Stc2 | SWGGLCSNL |
|  | Tada3 | SSASRRLLV |
| MC38 peptide pool 8 | Taf5l | FMSCNLLLV |
|  | Taf5l | VQFMSCNLL |
|  | Tmem106c | VVLDVTASL |
|  | Vps13d | RALSGLEPF |
| MC38 peptide pool 9 | Yipf4 | VAYGQVLAV |
|  | Yipf4 | LAVIGYSLL |
|  | Zfand4 | AALHRLLRV |
|  | Zfp644 | LGHLHRAGL |

**Supplementary Table S3**: List of 4T1 neoepitopes

| **Gene** | **Peptide sequence** |
| --- | --- |
| Gen1 | IPHNPRVAVKTTNNLVMKNSVCLERDS |
| Polr2a | LAAQSLGEPATQITLNTFHYAGVSAKN |
| Tmtc2 | QGVTVLAVSAVYDIFVFHRLKMKQILP |
| Zfr | AHIRGAKHQKVVTLHTKLGKPIPSTEP |
| Cept120 | ELAWEIDRKVLHQNRLQRTPIKLQCFA |
| Malt1 | FLKDRLLEDKKIAVLLDEVAEDMGKCH |
| Wdr11 | NDEPDLDPVQELIYDLRSQCDAIRVTK |
| Kbtbd2 | DAAALQMIIAYAYRGNLAVNDSTVEQL |
| Adamts9 | KDYTAAGFSSFQKLRLDLTSMQIITTD |
| Pzp | AVKEEDSLHWQRPEDVQKVKALSFYQP |
| Gprc5a | FAICFSCLLAHALNLIKLVRGRKPLSW |
| Enho | MGAAISQGAIIAIVCNGLVGFLL |
| Dmrta2 | EKYPRTPKCARCGNHGVVSALKGHKRY |
| Rragd | SHRSCSHQTSAPSPKALAHNGTPRNAI |
| Zzz3 | KELLQFKKLKKQNLQQMQAESGFVQHV |
| Ilkap | RKGEREEMQDAHVSLNDITQECNPPSS |
| Cenpf | RVEKLQLESELNESRTECITATSQMTA |

**Supplementary Fig. 1. (A)** PCR quantification of neomycin DNA relative to an internal standard is proportional to the presence of 4T1-pCMV cells. **(B)** Calibration curve of relative expression of neomycin DNA versus 4T1-pCMV number in mouse lungs. Linearity was confirmed from 10^3^ to 2 x 10^6^ cells (R^2^ = 0.975).

**Supplementary Fig. 2. (A)** Cytokines detected in parental and CSF1^-/-^ 4T1 cell culture supernatants (n = 2 spotted duplicates per analyte). Fold change mRNA expression levels of **(B)** 4T1 CSF1^-/-^ cells, and **(C)** MC38 CSF1^-/-^ cells relative to the parental control line. Only fold changes ≥ 2 relative to the parental cell line are shown.

**Supplementary Fig. 3.** In vivo tumor growth of (**A-B**) MC38 parental tumors, and (**C**) MC38 CSF1 -/- tumors treated with either vaccine, anti-PD-L1 (200 ug, IP) or combination therapy.

**Supplementary Fig. 4. (A)** Statistical analysis of IFNy ELISPOT assay of mice bearing 4T1 tumors following treatment with three doses of 500 ug CSF1R antibody. Splenocytes were harvested and incubated with 4T1 neoepitopes, and three TWIST1 25-mer peptides (n = 4 mice per group). **(B)** IFNy ELISPOT assay of mice bearing 4T1 tumors following treatment with Ad-Twsit vaccine.
